# Supplementary material for: Variation between hospital caesarean delivery rates when Robson’s classification is considered: An observational study from a French perinatal network
Source: PLoS One. 2021 Aug 20;16(8):e0251141. doi: 10.1371/journal.pone.0251141 (PMC8378683; doi:10.1371/journal.pone.0251141)
Supplement: Attachment — Submitted filename: review.docx [file pone.0251141.s002.docx]

Dear Editors,

I would like to thank you for the opportunity to review the manuscript titled “Variation between hospital caesarean delivery rates when Robson's classification is considered: an observational study from a French perinatal network” by Thibaud Quibel, M.D and colleagues.

The aim of the study was to examine the contribution of maternal socioeconomic and clinical characteristics and hospital organizational factors to the variation in cesarean delivery rates when considering Robson’s ten-group classification system.

This study was a prospective, observational, population-based study including all deliveries after viability (>24 weeks) at the 10 hospitals of the French MYPA perinatal network in the Paris area.

1) Upon review of the Robson’s criteria and the literature published concerning the topic, I agree with the authors that considerably relevant factors have not been considered when examining this tool to compare variations in cesarean rates. There have been multiple papers that have demonstrated that maternal and hospital/ system organizational factors clearly impact those calculations and cannot be ignored. If this system/tool is used for any quality indicators to effect change, these important factors should be factored into the score. As a result, I find the paper to be truly relevant and applaud the authors for the development of the concept, design and well thought out statistical plan.
2) The methods are well designed, and the statistics are explained and planned appropriately. I appreciate the sensitivity analysis excluding hospital A, which carried 25% of the deliveries and surely would influence the data given that it was a high-risk hospital. I am not surprised from the findings and results, which make complete sense. All of which are compatible with current high quality obstetrical data.
3) I wonder if the authors believe that the lower risks hospitals which did not have a NICU had lower cesarean rates because perhaps those mothers were transferred out? This would be a nice discussion point.
4) I also wonder why diabetes was not available as an obstetrical variable that may influence cesarean rates. As gestational and pregestational diabetes effect a significant proportion of pregnant women, that variable is hard to leave out. It is listed as a limitation, but the authors do not explain why that variable was left out.
5) With respect to institutional factors, I believe that private and public health and midwife to MD ratios are extremely relevant and appreciate those results. They make complete sense. These results speak to the experience of the providers and the true level of acuity each hospital is comfortable with or equipped to handle. Line 281-4 imply that it is more preference base (financial incentives, fear of litigation, etc) when in fact, these providers may not feel that way but perhaps, the hospitals are not structured and approved for certain medical conditions and obstetrical complications. Personnel and finance are vastly different for a level 1 vs. level 3 hospital. I think those lines can be rewritten to reflect professional objective measures.

This is an excellent paper and I recommend approval, however if possible, if the authors could add the diabetes data. If not, please explain why data is not available more clearly in the discussion. In addition, a brief rewrite of lines 281-4 to reduce subjectivity and last, if the lack of NICU and lower cesarean rate data could be explained in the discussion as well.
